# Beta-Endorphin 1–31 Biotransformation and cAMP Modulation in Inflammation

**DOI:** 10.1371/journal.pone.0090380

**Published:** 2014-03-11

**Authors:** Naghmeh Hajarol Asvadi, Michael Morgan, Herath M. Herath, Amitha K. Hewavitharana, P. Nicholas Shaw, Peter J. Cabot

**Affiliations:** School of Pharmacy, The University of Queensland, Brisbane, Queensland, Australia; Università di Napoli Federico II, Italy

## Abstract

A large body of evidence now exists for the immune cell expression, production, and the release of beta-endorphin (BE 1–31) within inflamed tissue. The inflammatory milieu is characterised by increased acidity, temperature and metabolic activity. Within these harsh conditions BE 1–31 is even more susceptible to increased enzymatic degradation over that of plasma or other non-injured tissue. To elucidate the biotransformation pathways of BE 1–31 and provide an insight to the impact of inflamed tissue environments, BE 1–31 and three of its major N-terminal fragments (BE 1–11, BE 1–13 and BE 1–17) were incubated in inflamed tissue homogenates at pH 5.5 for 2 hrs. In addition, the potency of BE 1–31 and five main N – terminal fragments (BE 1–9, BE 1–11, BE 1–13, BE 1–17, BE 1–20) was assessed at mu-opioid receptors (MOR), delta-opioid receptors (DOR), and kappa-opioid receptors (KOR). Opioid receptor potency was investigated by examining the modulation of forskolin induced cAMP accumulation. The majority of the N-terminal fragment of BE 1–31 had similar efficacy to BE 1–31 at MOR. The shortest of the major N-terminal fragments (BE 1–9), had partial agonist activity at MOR but possessed the highest potency of all tested peptides at DOR. There was limited effect for BE 1–31 and the biotransformed peptides at KOR. Major N-terminal fragments produced within inflamed tissue have increased presence within inflamed tissue over that of the parent molecule BE 1–31 and may therefore contribute to BE 1–31 efficacy within disease states that involve inflammation.

## Introduction

Beta-endorphin (BE 1–31) is an endogenous opioid peptide that has been shown to have important roles in pain, immune system function [1], [2], reward [3], and stress [4]–[6]. BE 1–31 is primarily produced in the pituitary gland and the brain but can also be synthesised and released by leukocytes [7], [8]. BE 1–31 is derived from its precursor pro-opiomelanocortin by an enzymatic process [9], [10]. During inflammation, the production of BE is increased in leukocytes and it is subsequently released in inflamed tissue after which it undergoes rapid biotransformation [11]–[13]. Therefore, the role of opioid peptide metabolites in peripheral analgesia is of paramount consideration in understanding peripheral opioid action. There is variability in the reported literature in regards to clearance, distribution and half-life of BE 1–31 [14], [15], which can be attributed substantially to the species studied, anatomical location and tissue source being examined.

The metabolism of BE 1–31 has previously been examined in rat brain [16]–[18], cultured aortic endothelial cells [19], human T cells, thymoma cell line [20], [21], human plasma [22], and human pituitary [23]. A recent study in our laboratory has identified biotransformed fragments of BE 1–31 in rat serum, rat inflamed tissue, and following tyrosine hydrolysis in media [24]. This study demonstrated that the hydrolytic metabolism of BE 1–31 in homogenised inflamed tissue was faster than in serum and trypsin incubation. The rate of metabolism of BE 1–31 at pH 5.5 was also higher than the rate of metabolism of BE 1–31 at pH 7.4. Within BE 1–31 the amino acids identified as the most susceptible for hydrolytic degradation were; (Tyr^1^–Gly^2^), (Lys^9^-Ser^10^), (Leu^17^-Phe^18^-Lys^19^-Asn^20^), (Lys^24^-Asn^25^), (Lys^28^-Lys^29^-Gly^30^-Gln^31^) [24]. Further investigation of BE 1–31 biotransformation in inflammatory disease may provide an insight into key fragments with different pharmacological actions unique to inflamed tissue.

Peripheral analgesia during inflammation has been demonstrated to result from the action of endogenous opioid peptides on opioid receptors located on sensory neurons [2], [11], [25], [26]. These opioid receptors consist of three major opioid receptor families, namely: μ opioid receptor (MOR), κ opioid receptor (KOR), and δ opioid receptor (DOR) [27], [28], all of which belong to the G protein coupled receptor family and are expressed not only within the central nervous system but also on peripheral sensory nerve terminals. BE-1–31 is a non-selective agonist with the highest affinity for MOR and DOR [25], [28]. Opioid peptides binding to opioid receptors results in the inhibition of adenylate cyclase, responsible for conversion of ATP to cAMP [29], [30]. The inhibition of cAMP prevents neurotransmitter release at the site of inflammation, and is then implicated in the production of analgesia [31]. Hence, measuring intracellular cAMP production is a useful tool for investigating the comparative potency of opioid peptides upon opioid receptors [26].

In this study we further examined the biotransformation of BE 1–31 and fragments (BE 1–9, BE 1–11, BE 1–13, BE 1–17, and BE 1–20) in homogenised inflamed tissue to elucidate the biotransformation pathways of major metabolites and investigated the opioid pharmacological action of these fragments on cAMP inhibition.

## Materials and Methods

Animal ethics approval was obtained from the University of Queensland Animal Ethics Committee in accordance with the National health and Medical research Guidelines on the use and maintenance of animals. Rats were housed on a 12 hr dark/light cycle with access to water and food *ad libitum*. Inflamed paw tissue was obtained from male Wistar rats. To induce inflammation, rats received intra-plantar injections of Freund's complete adjuvant (0.15 ml) under brief isoflurane (5%) anaesthesia. After 6 days, rats were euthanised and the inflamed tissues were immediately surgically removed.

### Materials

DMEM (Dulbecco's Modified Eagle Medium) and FBS (Fetal Bovine Serum) were purchased from PAA Laboratories GmbH, Australia. Trypsin-EDTA (0.25%), G418 sulfate and Versene (tetrasodium ethylenediaminetetraacetate) were supplied from Invitrogen, Mt Waverly, Australia. BE1–31, BE 1–20, BE 1–17, BE 1–13, BE 1–11, BE 19–31, BE 20–31, BE 29–31 were purchased from Mimotopes Pty. Ltd, Australia. Purity was greater than 95% for all peptides. were obtained as previously and purity was greater than 95% for all peptides.

Stimulation buffer (pH 7.4) containing 0.1% bovine serum albumin (BSA), 0.5 mM 3-isobutyl-1-methylxanthine (IBMX) and 5 mM 4-(2-hydroxyethyl)-1-piperazineethanesulfonic acid (HEPES) and Lysis/detection buffer (pH 7.4) containing 0.1% BSA, 0.3% Tween 20 (10%) and 5 mM HEPES in Milli-Q water were freshly prepared. BSA and Tween 20 were obtained from Research Organics, Cleveland, OH, USA. IBMX, SNC-80 (4-[(*R*)-[(2*S*,5*R*)-4-allyl-2,5-dimethylpiperazin-1-yl](3-methoxyphenyl)methyl]-*N*,*N*-diethylbenzamide) were purchased from Alexis Biochemicals, Switzerland, U50,488, 3-amino-propyltriethoxysilane (APTS), 2-(3,4-dichlorophenyl)-N-methyl-N-[(1R,2R)-2-(1-pyrrolidinyl)cyclohexyl]acetamide were supplied by Enzo Life Sciences, Inc, NY, USA. and forskolin (FSK) ((3R,4aR,5S,6S,6aS,10S,10aR,10bS)-6,10,10b-trihydroxy-3,4a,7,7,10a-pentamethyl-1-oxo-3-vinyldodecahydro-1H-benzo[f]chromen-5-yl acetate) were also obtained from Enzo Life Sciences, Inc, NY, USA. HEPES, Hank's balanced saline solution (HBSS), naloxone and sodium bicarbonate were supplied by Sigma, St. Louis, MO, USA. The determination of cAMP was performed using an Alphascreen cAMP assay kit (code 6760625M). Half-area 96 well microplates and microplate press-on adhesive sealing film were purchased from PerkinElmer (MA, USA).

### Cell culture

HEK 293 (Human Embryonic Kidney) cells independently and stably expressing MOR, DOR, KOR were used to examine opioid potency on the accumulation of cAMP. HEK293 cells were transfected with pcDNA3 containing FLAG-MOR using Lipofectamine 2000 (Invitrogen Australia Pty Ltd, Mulgrave, Victoria, Australia). Stable FLAG-MOR cells were selected using geneticin. Immunohistochemistry and western blotting were used to verify FLAG-MOR expression. Plasmids containing the cDNA encoding the murine FLAG-epitope-tagged μ-opioid receptor (FLAG-MOR) [32], A similar process was conducted for DOP and KOP-HEK cell lines. Passage numbers for HEK-MOR cells were 38–53. Passage numbers for HEK-DOR cells were 15–30, and passage numbers for HEK-KOR cells were 25–36.

### Analysis of metabolites of endogenous opioid peptides

BE 1–31, BE 1–17, BE 1–13, BE 1–11 metabolism in rat inflammatory tissue homogenates. Inflamed tissue was homogenised in isotonic MES buffer (pH 5.5, 2-(N-morpholino) ethanesulfonic acid); Sigma (St. Louis, MO, USA) at a ratio of 1 g tissue to 10 ml of buffer. The tissue homogenate was then centrifuged for 10 min (4°C) at 1600 RCF and the supernatant collected. Each peptide was added separately to the tissue homogenate to a resultant final concentration of 115 µM of peptide optimised for analysis [24]. Samples (100 µl) of the peptide/tissue homogenate mixture were collected at intervals during a 120 min incubation. The protein content of the samples was precipitated by the addition of acetonitrile (200 µl) and the sample vortex mixed for 1 min. Samples were incubated for 20 min at room temperature followed by 15 min centrifugation at 12 500 RCF at room temperature. The supernatant was transferred to a fresh microcentrifuge tube and dried under nitrogen on a heating block at 37°C. Milli-Q water (75 µl) was added to each dried sample. Samples were then transferred to LC/MS vial inserts for subsequent analysis.

### Liquid chromatography

An Agilent binary HPLC composing of an Agilent 1100 LC pump, an Agilent 1100 well plate auto-sampler (Agilent Technologies, Santa Clara, CA, USA) and a Jupiter 5 μ (C4, 300 A, 150×2.00 mm, 5 μ) RP HPLC column (Phenomenex, Torrance, CA, USA) were used for separation. The mobile phase flow rate was 200 µl/min and the volume of injection was 20 µL. The separations were performed using a binary solvent gradient; water (Milli-Q; Millipore Corporation, Bedford, MA) which contained 0.1% v/v formic acid was used as the solvent A and acetonitrile (Merck KGaA, Darmstadt, Germany) which contained 0.1% v/v formic acid (Nuplex industries Pty Ltd. (NSW, Australia) was used as the solvent B. The chromatographic separation was achieved using the gradient shown in Table 1.

**Table 1:** Chromatographic analysis gradients used for solvents A and B in the separation of beta-endorphin fragments.

Time (min)	A%	B%
0	100	0
3	100	0
33	50	50
38	0	100
40	0	100
43	100	0
53	100	0

### Mass spectrometry

An API 3000 tandem mass spectrometer equipped with a turbo ion spray was used as the detector. Detection was performed in total ion current (TIC) mode (MS1/Q1), in positive mode, within the range 200–3000 amu. The data were acquired and processed by Analyst 1.5 software (Applied Biosystems, Foster city, CA, USA). Mass spectra were obtained using: declustering potential (DF) of 16 V, focusing potential (FP) of 120 V, Entrance potential (EP) of 10 V, and Ion spray voltage (IS) of 5000 V. Structure identity of fragments was performed using the respective mass/charge (m/z) values of each peptide metabolite. Direct flow injections were carried out using Model 11 plus Harvard Syringe pump (Harvard Apparatus Inc, Holliston, MA, USA).

### Mean Residence Time Calculations for Identified Fragments

MRT represents the average time that a molecule resides in a particular environment and can be calculated as follows:
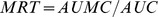
Where the AUMC is area under the first moment curve and AUC is area under the concentration-time curve. The areas can be expressed in the following manner where *C(t)* is compound concentration at time *t* [33], [34]. In the following calculations peptide peak intensity was taken to represent the respective peptide concentration.
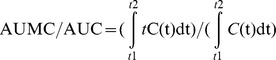
Relative MRT values were calculated from the ratio of the respective MRT of each fragment to the MRT of BE 1–31.

### cAMP alphascreen assay

Cells were cultured in DMEM containing 10% w/v FBS and 0.1% w/v G-418. Cells grown to near confluence (in 75 cm^3^ plates) were used for the cAMP assay. Cells were washed with PBS and detached by incubating at 37°C for 5 min with Versene. Cells were then diluted with PBS (5 mL) and centrifuged at 280 g for 5 min at 23°C. Separated cells were reconstituted in stimulation buffer. A mixture of opioid peptide and FSK was prepared in stimulation buffer (final concentration of BE 1–31 and its fragments in well was 1 µM to 0.3 nM in half log intervals). The mixture of opioid peptides and forskolin were plated on 96 well plates. A working solution of anti-cAMP acceptor beads (0.4 units/µL) was prepared from a stock solution provided in the assay kit. The counted cells were mixed with prepared solution of acceptor beads and were added to each well containing FSK and opioid peptides for 30 min. Streptavidin donor beads/biotinylated cAMP detection mixture containing 1unit/25 µL of biotinylated cAMP and 1unit/25 µL of donor beads in Lysis/detection buffer was prepared and incubated in dark conditions for at least 30 min prior to use. The EnSpire Multilabel reader from PerkinElmer, MA, USA was used for the Alphascreen analysis. The opioid receptor nature of cAMP inhibition was examined by pre-incubation with the non selective opioid antagonist naloxone, Naloxone (100 µM) was added to cell and bead mixtures 30 min prior adding of FSK and opioid peptides.

### Statistical analysis

Data calculations and statistical analysis were performed using Prism software version 6 (GraphPad Software Inc., CA, USA). The concentration-response curves were plotted using one-site curve fitting in the Prism software using nonlinear regression analysis tools in Prism. Confidence intervals (95%) were implemented for comparison of IC50 of BE 1–31 and each fragment. The comparison between naloxone treated and naloxone untreated groups were performed using one-way ANOVA followed by Newman-Keuls post-test. Significance is denoted by * (*P*<0.05).

## Results

### Biotransformation of BE 1–31 in homogenized inflamed tissue at pH 5.5

**Figure 1:**
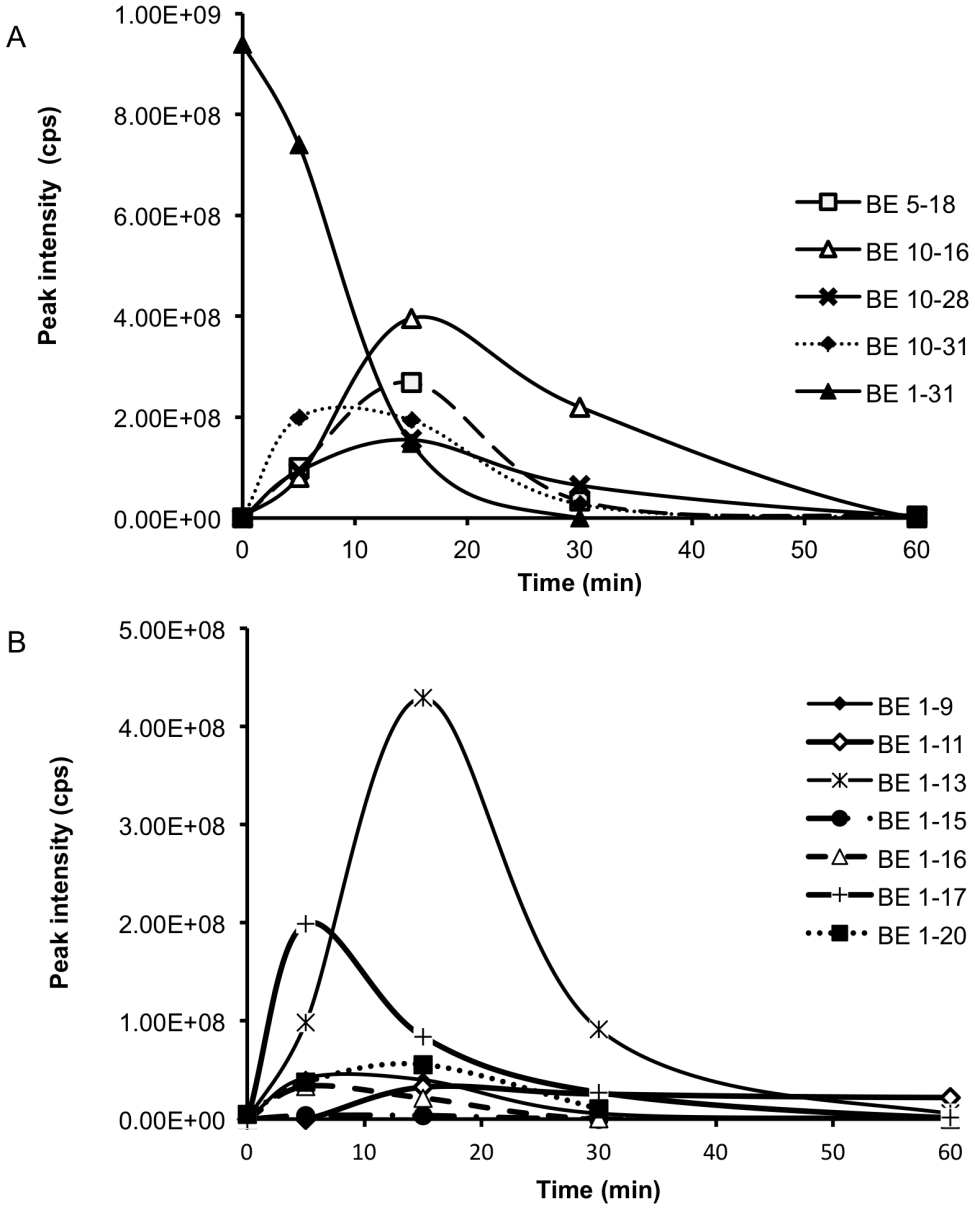
Biotransformation of BE 1–31 in inflamed tissue at pH 5.5 at 37°C over 60 minutes. **A**. The production and degradation of major intermediate fragments during the incubation of BE 1–31 at pH 5.5 at 37°C over 60 minutes. The peak area of each fragment was determined at each time point and plotted. **B**. The production and degradation of main N-termianl fragments during the incubation of BE 1–31 at pH 5.5 at 37°C over 60 minutes.

BE 1–31 was incubated in homogenised inflamed tissue in MES buffer at pH 5.5 indicative of tissue acidosis inflamed tissue *in vivo* and was detected using LC/MS with a retention time of 25.8 min. The peak intensity of BE 1–31 reduced rapidly such that only 7.5% was observed at 15 min (Figure 1, A). A total of 29 metabolites were identified following 2 h of incubation of BE 1–31 in inflamed tissue at pH 5.5. The hydrolysis pattern of BE 1–31 and the observed mass/charge values of the fragments are shown in Table S1. MRT and relative MRT for BE 1–31 and the respective peptide fragments were calculated and are listed in Table S1. BE 1–16, BE 1–17, BE 1–18, BE 1–20, BE 18–31 and BE 19–31 appeared after 2 minutes of incubation of BE 1–31 in inflamed tissue at pH 5.5 indicating susceptibility to enzymatic cleavage between Leu-17 and Phe-18, and between Phe-18 and Lys-19. BE 1–13 was one of the major metabolites detected after 5 minutes of incubation of BE1–31 and demonstrated a high relative peak intensity compared to other metabolites at 15 min. The time profiles of the N-terminal fragments of BE 1–31 are shown in Figure 1, B, over a 1 hr period. BE 1–13 and BE 1–17 displayed higher peak intensities than the other N-terminal peptide fragments during incubation of BE 1–31 inflamed tissue homogenates. Moreover, BE 1–13 was present within inflamed tissue homogenates for over 1 hour during the incubation period (Figure 1, B). C-terminal metabolites such as BE 19–31, BE 20–31 and intermediate metabolites (including BE 10–16, BE 20–24, BE 20–27, and BE 20–28) were present had a higher relative MRT inflamed tissue homogenates in comparison to N-terminal metabolites (Table S1). BE 10–16 was observed in homogenised inflamed tissue for over 60 minutes with a high peak intensity relative to other intermediate metabolites (Figure 1, A).

### Biotransformation of BE 1–17 in homogenized inflamed tissue at pH 5.5

**Figure 2:**
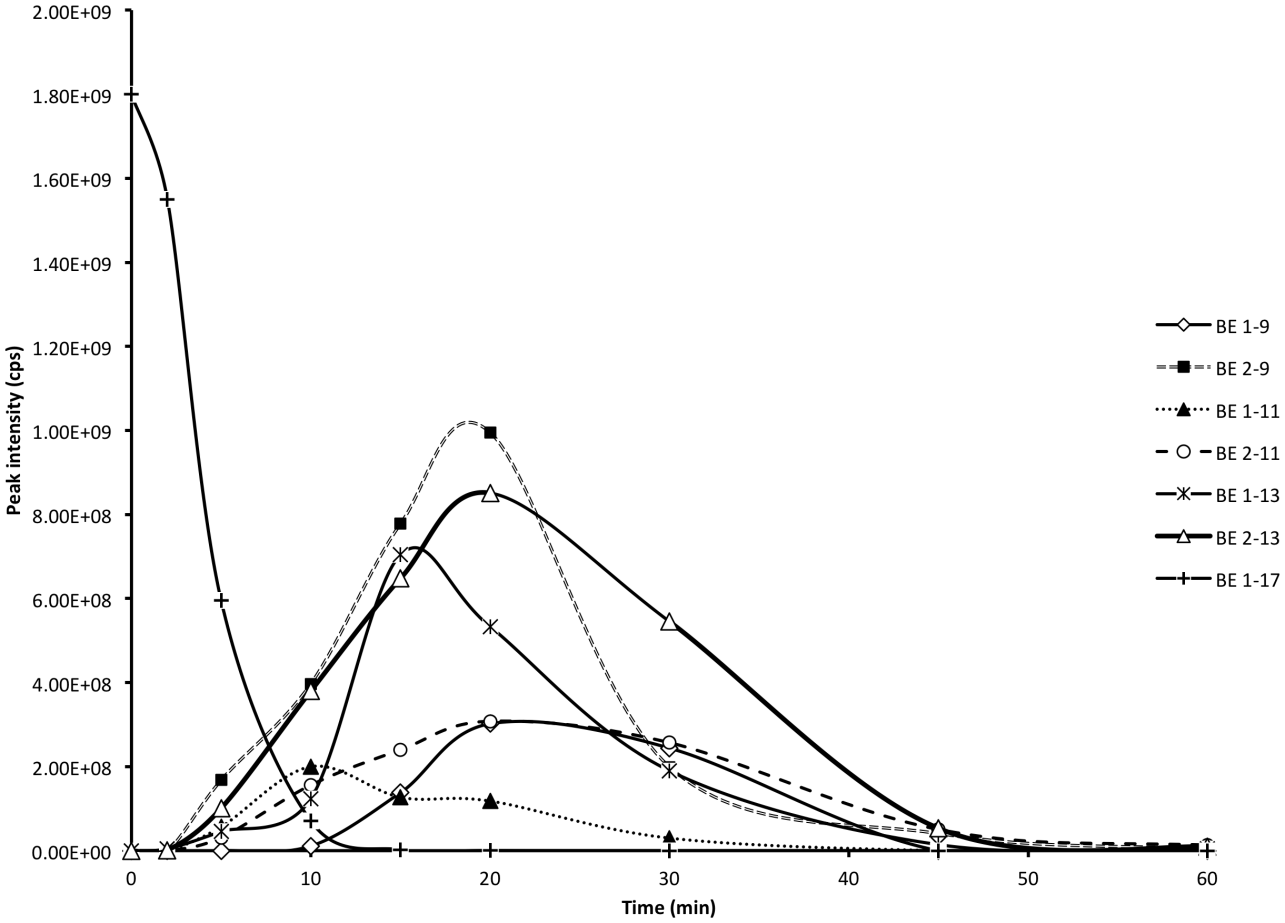
Biotransformation of BE 1–17 in inflamed tissue at pH 5.5 at 37°C over 60 minutes. The production and degradation of BE 1–9, BE 2–9, BE 1–11, BE 2–11, BE 1–13, BE 2–13 during incubation of BE 1–17 in inflamed tissue at pH 5.5 at 37°C over 60 minutes. The peak area of each fragment was determined at each time point and plotted.

**Figure 3:**
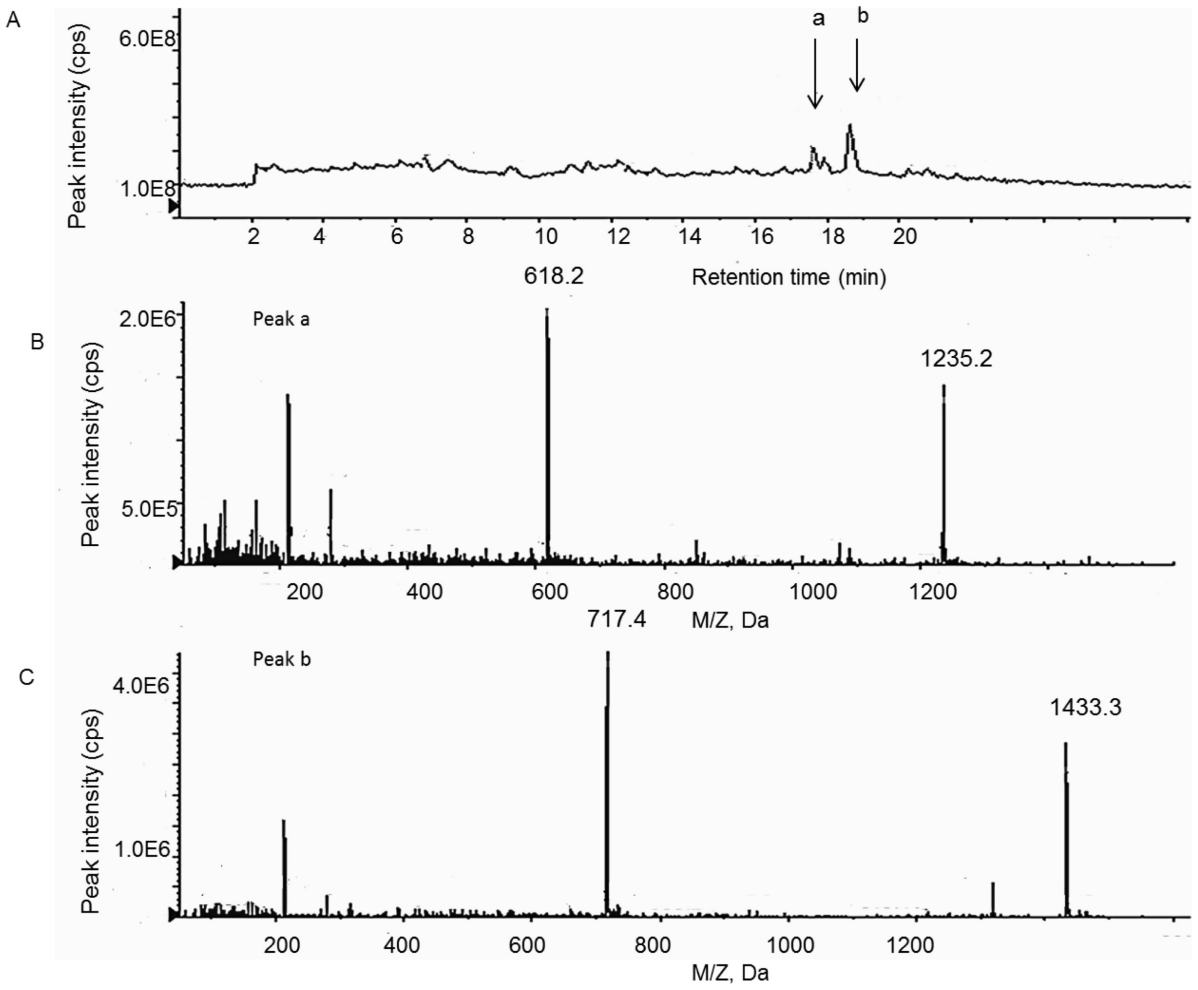
Biotransformation of BE 1–17 in homogenised inflamed tissue at 37°C. **A**. The TIC spectra of BE 1–17 and its fragments detected after 45 minutes incubation at 37°C with inflamed tissue at pH 5.5. **B**. Peak **a** at retention time of 17.6 minutes is BE 1–11 with following observed mass/charge values [M+H]^+1^:1235.2 and [M+H]^+2^: 618.2. **C**. Peak b at retention time of 18.6 minutes is BE 1–13 with following observed mass/charge values [M+H]^+1^:1433.3 and [M+H]^+2^: 717.4.

BE 1–31 underwent enzymatic cleavage between Leu-17 and Phe-18 to produce BE 1–17. BE 1–17 was hence examined to elucidate its stability and biotransformation in inflamed tissue homogenates at pH 5.5. The peptides identified from the biotransformation of BE 1–17 are listed in Table S2. BE 1–17 was detected at a retention time of 24.5 min and was hydrolysed to undetectable levels after a 15 min incubation period (Figure 2). BE 1–16, BE 1–15, BE 2–17, and BE 3–17 were detectable after 2 min incubation of BE 1–17. BE 2–17, BE 3–17, BE 2–16, and BE 3–16, although identified at 2 min, were undetectable after 5 min. BE 1–11, BE 1–13 (Figure 3), and des-tyrosine metabolites such as BE 2–9, BE 2–13, and BE 2–17 were major metabolites from the incubation of BE 1–17 in inflamed tissue homogenates. BE 2–9 was evident prior to the appearance of BE 1–9 in the incubation matrix, with a greater peak intensity (Figure 2). BE 2–13 and BE1–13 were detected at the earliest time point of 2 min (Figure 2). BE 2–11 was another major metabolite of BE 1–17 with higher relative peak intensity than BE 1–11 (Figure 2).

### Biotransformation of BE 1–13 in homogenised inflamed tissue at pH 5.5

**Figure 4:**
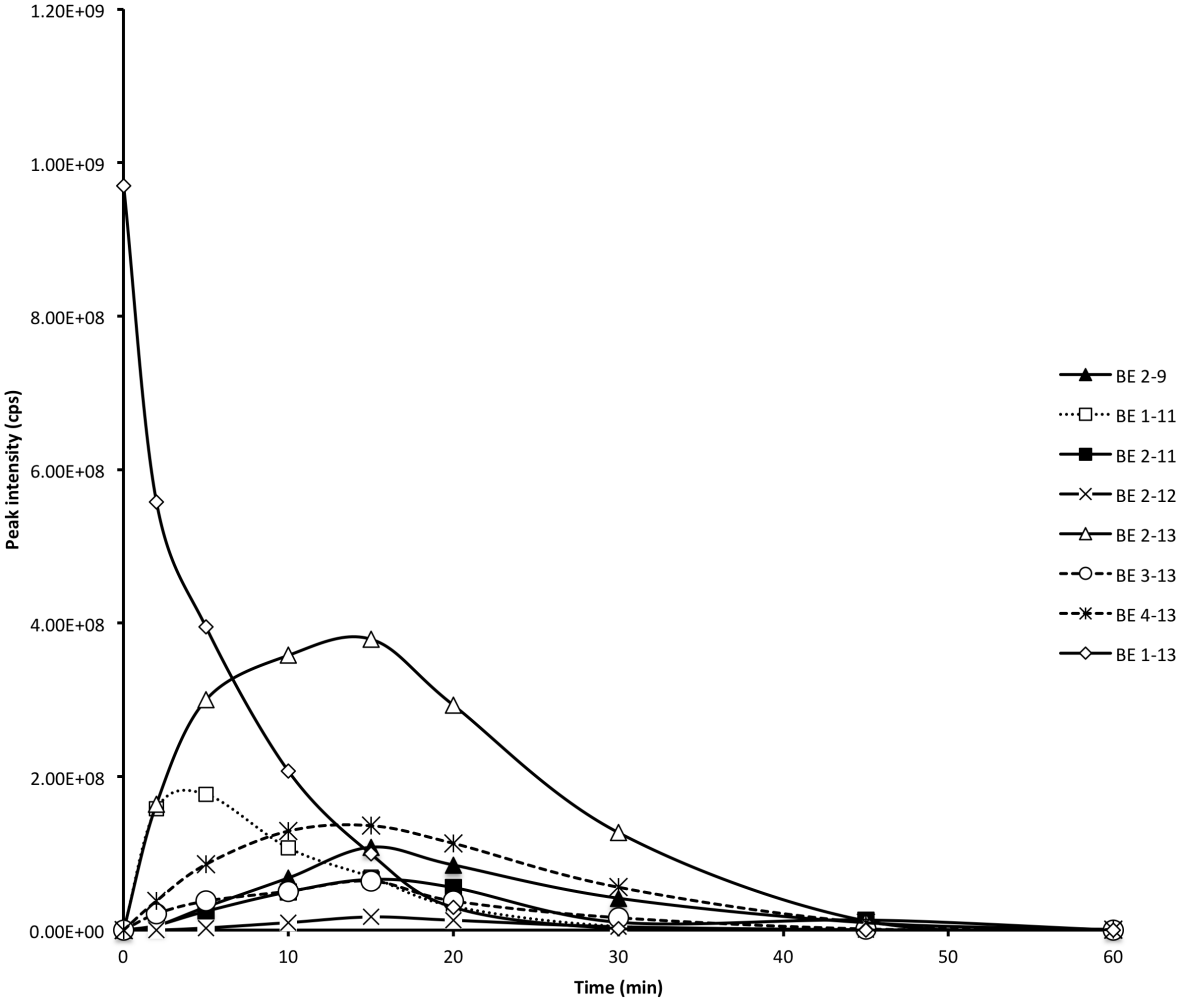
Biotransformation of BE 1–13 in inflamed tissue at pH 5.5 at 37°C over 60 minutes. The production and degradation of fragments during incubation of BE 1–13 in inflamed tissue at pH 5.5 at 37°C over 60 minutes. The peak area of each fragment was determined at each time point and plotted.

BE 1–13 was a major fragment observed during the incubations of BE 1–31 and BE 1–17 in homogenised inflamed tissue, arising from enzymatic cleavage between Pro-13 and Leu-14. BE 1–13 was observed at retention time 18.4 min and it was detected in inflamed tissue homogenates for 30 min (Figure 4). BE 1–11 and BE 2–13 were both detected at the earliest time point examined (2 min) after incubation of BE 1–13 in inflamed tissue homogenates (Figure 4) and BE 2–13 appeared to be the principal metabolite of BE 1–13. BE 2–12, BE 3–13, BE 4–13, and BE 2–11 were produced following the detection of BE 1–11 and BE 2–13 (Figure 4). All the major metabolites with the exception of BE 1–11 reached their highest peak intensity after 15 minutes and were not present after 45 minutes (Figure 4). BE 1–11 reached its highest peak intensity after 5 minutes of incubation of BE 1–13 in inflamed tissue (Figure 4). The pattern of biotransformation of BE 1–13 is shown in Table S3. In contrast to the metabolism of BE 1–17, BE 2–11 was observed at a lower peak intensity than BE 1–11 in metabolism studies of of BE 1–13 in homogenised inflamed tissue at pH 5.5.

### Biotransformation of BE 1–11 in homogenised inflamed tissue at pH 5.5

**Figure 5:**
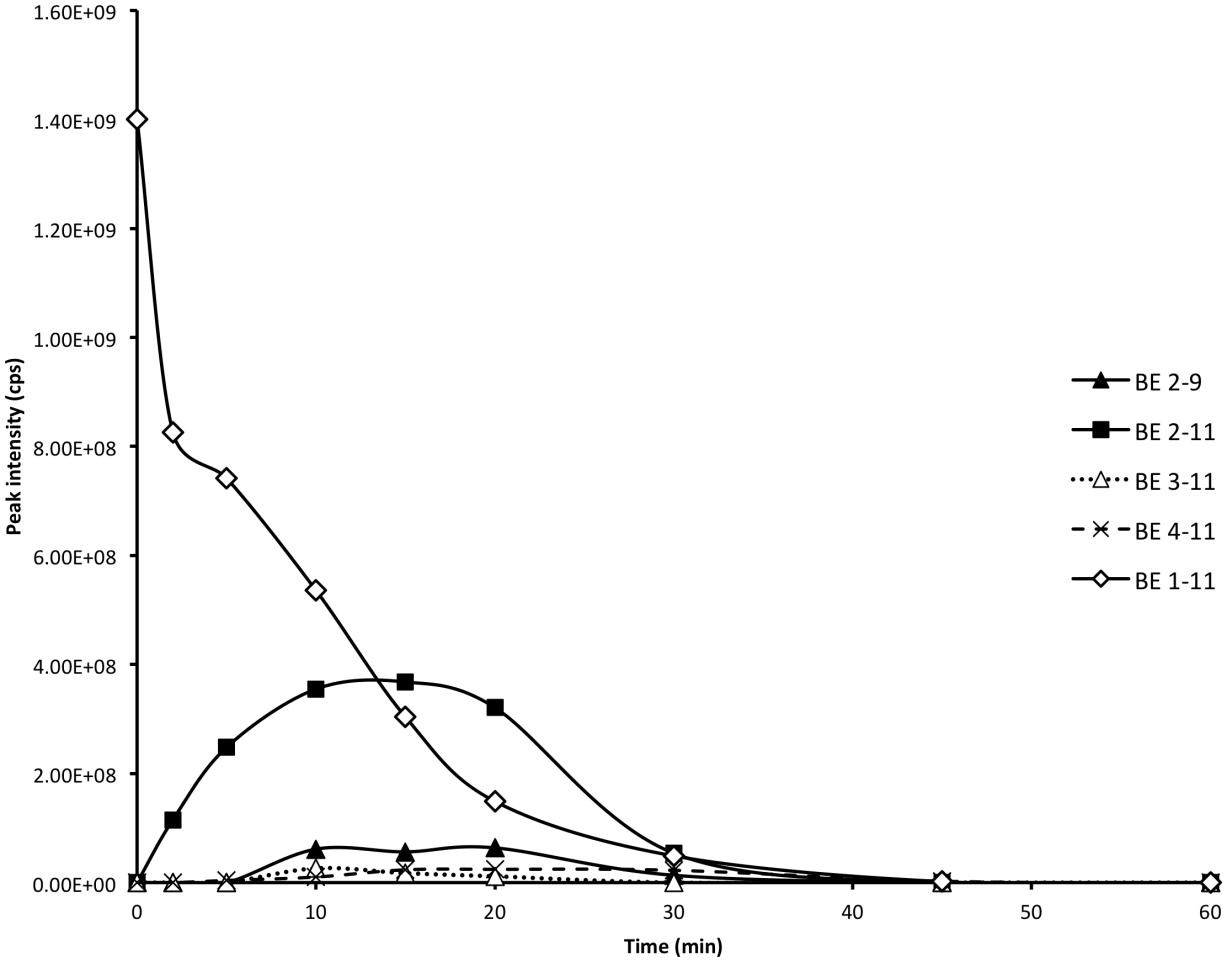
Biotransformation of BE 1–11 in inflamed tissue at pH 5.5 at 37°C over 60 minutes. The production and degradation of fragements during incubation of BE 1–11 in inflamed tissue at pH 5.5 at 37°C over 60 minutes. The peak area of each fragment was determined at each time point and plotted.

BE 1–11 was observed following the incubation of BE 1–31, BE 1–17, and BE 1–13, arising from enzymatic hydrolysis between Gln-11 and Thr-12. BE 1–11 was detected at a retention time of 17.4 min but was undetectable after a 30 min incubation period (Figure 5). The biotransformation of BE 1–11 in inflamed tissue homogenates revealed four major fragments: BE 2–11, BE 3–11, BE 4–11 and BE 2–9. The biotransformation pattern of BE 1–11 in inflamed tissue is depicted in Table S4. The N-terminal cleavage of BE 1–11 produced metabolites which were relatively more polar than the parent compound; BE 4–11 was observed at an earlier retention time (13.8 min). BE 2–11 was the major hydrolysis fragment with higher peak intensity compared with all of the other metabolites of BE 1–11. The peak intensity of BE 2–11 was observed to be approximately 4 times higher than that of BE 2–9 (Figure 5). BE 3–11 and BE 4–11 had lower peak intensities when compared to BE 2–9. The metabolites of BE 1–11 reached their highest peak intensity after 15 minutes of incubation of BE 1–11 in inflamed tissue at pH 5.5 (Figure 5).

### Evaluation of concentration dependent cAMP inhibition by BE 1–31, BE 1–20, BE 1–17, BE 1–13, BE 1–11, and BE 1–9 in HEK cells expressing MOR

The concentration-dependent effects of selected N-terminal fragments of BE 1–31 on the inhibition of cAMP accumulation (stimulated by FSK) in HEK 293 cells expressing MOR are shown in Figure S1. Fentanyl is a full agonist at the MOR and was used as a positive control. The inhibition potency of BE 1–9 in the cAMP assay was less than those of the other N-terminal fragments tested (*P*<0.05). The IC_50_ values of fragments (BE 1–11, BE 1–13, BE 1–17, BE 1–20) was not significantly different to that of BE 1–31 (Table 2).

**Table 2:** BE fragments IC50 for the inhibition of forskolin induced cAMP in HEK-MOP cells.

Compound	IC 50	95% confidence interval
BE 1–9	4.3 nM	2.6 nM–7 nM
BE 1–11	21 nM	13 nM–34 nM
BE 1–13	8.4 nM	4.9 nM–14 nM
BE 1–17	6.5 nM	4.3 nM–9.8 nM
BE 1–20	5 nM	3.4 nM–7.2 nM
BE 1–31	24 nM	14.5 nM–41 nM
SNC 80	8.1 nM	4.8 nM–14 nM

**Figure 6:**
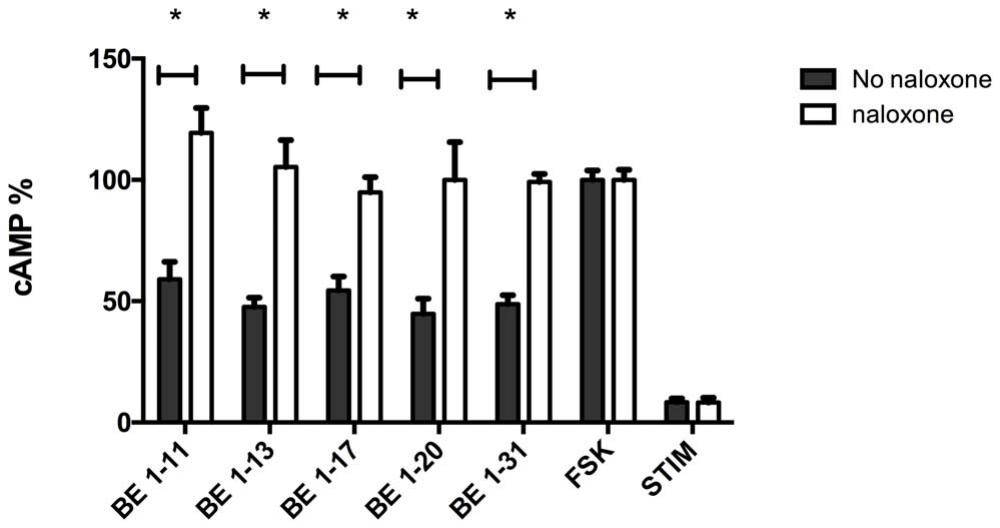
The effect of naloxone treated and naloxone untreated on the cAMP inhibition of 50–11, BE 1–13, BE 1–17, and BE 1–31 in HEK 293 cells expressing MOR. Naloxone (100 µM) was added to the cells (20000 cells/well) 30 min prior adding opioid peptide and FSK (100 µM) in order to block MOR. In naloxone treated cells cAMP levels were significantly higher than those in absence of naloxone (*P<0.05), one-way ANOVA, post-test newman-keuls multiple comparison test). Values represent mean ± SEM of at least three independent experiments. FSK: No opioid peptide. STIM: No opioid peptide and no FSK.

To confirm the effect of BE 1–31 and its fragments on the inhibition of cAMP production through activation of MOR, the HEK-MOR cells were pre-treated with naloxone. Naloxone is a non-selective opioid antagonist. The cAMP inhibition of BE 1–11, BE 1–13, BE 1–17, BE 1–20, and BE 1–31 were inhibited by naloxone (Figure 6).

### Evaluation of concentration dependent cAMP inhibition by BE 1–31, BE 1–20, BE 1–17, BE 1–13, BE 1–11, and BE 1–9 in HEK cells expressing DOR

The concentrations dependent effect of BE 1–31 and the selected N-terminal fragments on DOR mediated forskolin stimulated cAMP accumulation were studied using HEK-DOR cells (Figure S2). The DOR agonist, SNC80, was used as a positive control. All of the selected BE1–31 fragments at concentrations of 1 µM or greater were shown to be capable of inhibiting cAMP through DOR. There was no significant difference between any of the IC50 values of BE 1–9, BE 1–17, and BE 1–20. However, the IC50 values for BE 1–9, BE 1–13, BE 1–17, BE 1–20 were significantly different to that of the parent molecule (BE 1–31) (Table 3, *P*<0.05).

**Table 3:** BE fragments IC50 for the inhibition of forskolin induced cAMP in HEK-DOP cells.

Compound	IC 50	95% confidence interval
BE 1–11	18 nM	10 nM–32 nM
BE 1–13	59 nM	35 nM–0.1 µM
BE 1–17	25 nM	18 nM–34 nM
BE 1–20	55 nM	15 nM–0.2 µM
BE 1–31	57 nM	34 nM–95 nM
Fentanyl	5 nM	2.3 nM–11 nM

**Figure 7:**
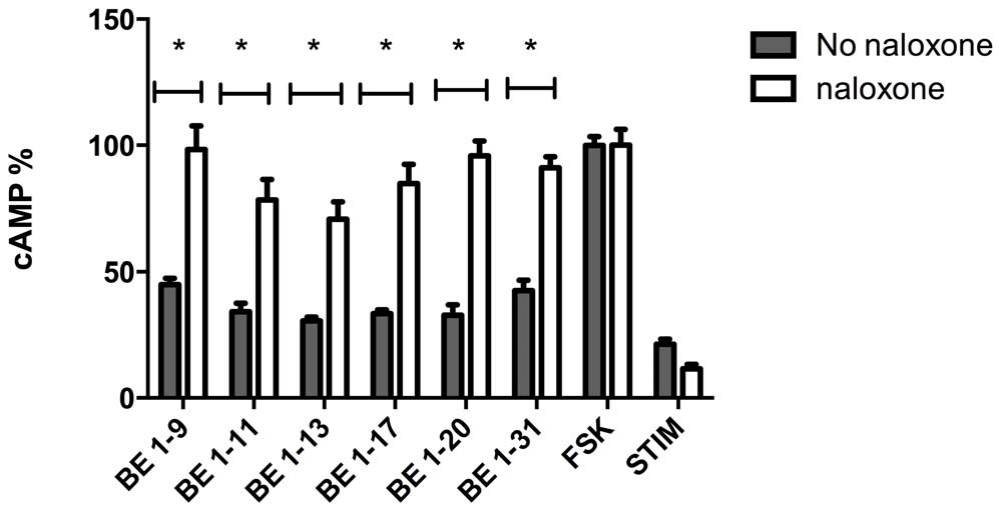
The effect of naloxone treated and naloxone untreated on the cAMP inhibition of 50–11, BE 1–13, BE 1–17, and BE 1–31 in HEK 293 cells expressing DOR. Naloxone (100 µM) was added to the cells (20000 cells/well) 30 min prior adding opioid peptide and FSK (50 µM) in order to block MOR. The opioid peptides inhibition of the accumulation of cAMP was blocked by pre-treatment with naloxone. In naloxone treated cells cAMP levels were significantly higher than those in absence of naloxone (*P<0.05), one-way anova, post-test newman-keuls multiple comparison test). Values represent mean ± SEM of at least three independent experiments. Values represent mean ± SEM of at least three independent experiments. FSK: No opioid peptide. STIM: No opioid peptide and no FSK.

To confirm the activity of BE 1–31 and fragments (BE 1–9, BE 1–11, BE 1–13, BE 1–17, BE 1–20) on DOR, the effect of BE 1–31 and selected fragments on the production of cAMP in pre-treated cells with naloxone was compared with the effect of BE 1–31 and its fragments on the production of cAMP in absence of naloxone. BE 1–31 and fragments that inhibition of cAMP through DOR were blocked by naloxone (Figure 7, *P*<0.05).

### The effects of BE 1–31, BE 1–20, BE 1–17, BE 1–13, BE 1–11, and BE 1–9 on the inhibition of forskolin-stimulated cAMP accumulation in HEK cells expressing KOR

**Figure 8:**
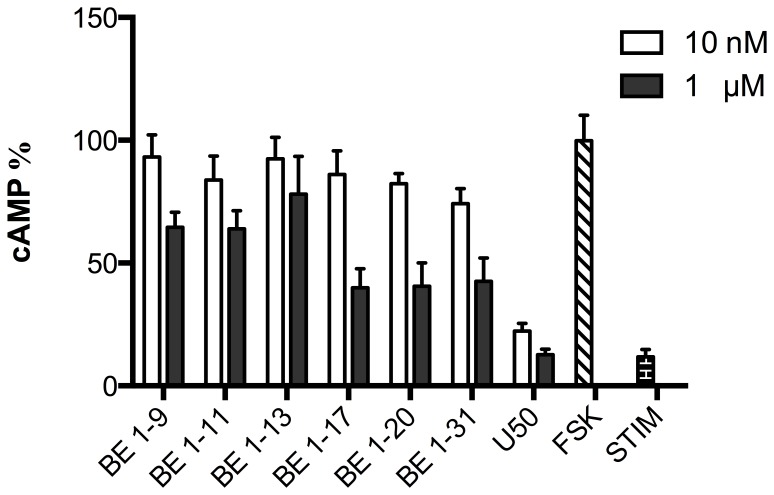
Screening of BE 1–31 and its metabolites on cAMP inhibition in HEK 293 cells expressing KOR (10 nM and 1 µM). The screening of BE 1–31 and its fragments were performed by using 1000 cells/well with FSK (300 µM) to investigate the effect of BE 1–31 and its fragments on activation of KOP by measuring the level of cAMP. Values represent mean ± SEM of at least three independent. BE 1–31 and its fragments had limited activity at KOR modulation of cAMP.

High and low concentrations (1 µM and 10 nM) of BE 1–31 and selected N-terminal fragments (BE 1–9, BE 1–11, BE 1–17, BE 1–20) were examined for their modulation of the accumulation of cAMP in HEK cells expressing KOR (Figure 8). U50488H is a full agonist of the KOR and was used as a positive control (1 µM and 10 nM). BE 1–31 and selected fragments had limited activity upon cAMP inhibition through KOR. The IC_50_ values of BE 1–9, BE 1–11, BE 1–13 were greater than 1 µM (Figure 8).

## Discussion

BE 1–31 is released from immune cells within inflamed tissue to provide analgesia [7]. BE 1–31 is not a stable peptide and is susceptible to increased enzymatic degradation within the inflamed mileu of tissues. There is limited information on the relative contributions of BE fragments to analgesia in inflammatory pain [22], [35]. The cleavage of BE peptides is proposed to be largely due to aminopeptidases [36], angiotensin-converting enzyme (ACE), serine peptidases [22], insulin degrading enzyme [37], dipeptidyl peptidase III and dipeptidyl peptidase IV (DPP III, DPP IV) [38]. DPP IV has been shown to cleave at proline amino acids, like that found at position 13 in BE 1–31. Serine proteases have been shown to be responsible for cleavage of BE1–31 producing BE 1–19 and BE 20–31. Insulin degrading enzyme has been shown to produce BE 1–17 and BE 1–18 from BE 1–31 [37]. Moreover, enzyme activity has been shown to alter with the change in acidic pH environment [39], [40], consistent with that seen in inflamed tissue and in models of tissue acidosis [41], [42].

In this study, BE 1–31 was incubated in homogenised inflamed tissue, revealing 29 identified metabolites. The analyte extraction method reported above using acetonitrile as a protein precipitant, followed by LC-MS analysis, and hence was able to resolve a greater number of peaks compared to the previous methodology [24]. This is likely to result from an improved protein removal process and thereby reduced ionisation suppression in the MS detection of the peptides. Potential cleavage sites between the following amino acids in BE 1–31 were characterised as; Lys^9^-Ser^10^, Ser^10^-Gln^11^, Pro^13^-Leu^14^, Leu^17^-Phe^18^, Phe^18^-Lys^19^, Lys^19^-Asn^20^. The C-terminal metabolites eluted at a lower retention time, indicative of their higher polarity when compared to N-terminal metabolites. We also report herein a method for assessing the residence time (MRT) of peptide fragments within incubations using the application of statistical moment theory [34]. It is noted that C-terminal fragments displayed higher MRT than the N-terminal fragments of BE 1–31 implying their elongated persistence in inflamed tissue.

Similar to the current study, BE 1–16, BE 1–17, and BE 1–18 were detected as primary metabolites in the extracellular biotransformation of BE 1–31 in rat striatum and cerebrospinal fluid [35] implying amino acid sequences ^16^Thr-^17^Leu-^18^Phe-^19^Lys are potential site for enzymatic degradation of BE 1–31. BE 1–17, BE 1–18, BE 18–31 and BE 19–31 were also detected as major metabolites after metabolism of BE 1–31 in T cells, thymoma cells [20] and cultured aortic endothelial cells [19] confirming that^17^Leu-^18^Phe-^19^Lys which is connector area in structure of long peptide like BE 1–31 is more susceptible for enzymatic degradation.

BE 1–17, noted in our studies as one of the major BE1–31 fragments, was examined independently for its specific biotransformation pathway. The rate of hydrolysis (assessed using MRT data) at the C-terminus of BE 1–17 was greater than that of the enzymatic cleavage at N-terminus of that peptide over a 2 h incubation. BE 1–17 incubation with inflamed tissue homogenates yielded BE 1–16 and BE 1–15, BE 1–14, and BE 1–13 within the first 5 minutes of sampling. This demonstrated that BE1–17 undergoes rapid, sequential, C-terminal metabolism. BE 2–9, BE 2–13, and BE 2–11 were also observed as major secondary metabolites of the metabolism of BE 1–17. BE 2–9, as one of major hydrolysis products of BE 1–17, was evident prior to the appearance of BE 1–9 with a greater peak intensity - highlighting its potential formation from longer des-tyrosine fragments such as BE 2–17, BE 2–16, BE 2–13, BE 2–11. The conclusion that the BE 2–13 peptide was produced principally from BE 1–13 is supported by the observation the production of BE 2–13 increased coincidentally as the degradation of BE 1–13 increased. BE 2–11 was observed as a significant metabolite of BE 1–17 hydrolysis very possibly from a BE 1–11 precursor. BE 2–11 however, demonstrated higher peak intensity than BE 1–11 indicating that it may be produced from other peptides in addition to BE 1–11. In general, the *des*-tyrosine metabolites (BE 2–9, BE 2–11, BE 2–13) had greater peak intensities when compared to those of BE 1–9, BE 1–11, and BE 1–13.

It has previously been demonstrated that a number of the des-tyrosine hydrolysis metabolites of BE 1–17 may produce a range of biological effects. Wied *et al.* showed that BE 2–17 mimics certain defined effects consistent with that of neuroleptics [43]. Furthermore, both BE 2–16 and BE 2–17 were reported to increase natural killer cell activity. BE 2–16 and BE 2–17, acting as non-opioid fragments, increased lymphocyte natural killer cell activity via an unknown mechanism [44], with efficacy above that of opioid fragments such as BE 1–31 and BE 1–17. The peptides BE 2–16 and BE 2–9 were also shown to have amphetamine-like activity in rats delaying the extinction of pole-jumping avoidance behaviour and facilitated passive avoidance behaviours [43]. In addition, BE 2–9 increased the stereotypical sniffing response induced by amphetamine injection into nucleus caudatus [43]. BE 10–16, as a one of major intermediate hydrolysis metabolites which was identified in the biotransformation of BE 1–31 in homogenised inflamed tissue, has previously been shown to produce serotonin-like effects, inhibiting the behavioural effect of melatonin injected into the *nucleus accumbens* [43], [45].

BE 1–31, as an endogenous opioid peptide, has been shown to produce analgesia through the activation of opioid receptors during inflammation caused by tissue damage, stress or infection [7]. BE 1–31 has been suggested to produce its analgesic effects predominantly through the MOR and DOP [46]. Studies have also shown that N-terminal tyrosine is necessary for high affinity binding to classical opioid receptors as a structural determinant for analgesic activity [29]. Therefore, here we investigated five major N-terminal fragments for their potential as agonists at opioid receptors in a number of transformed cell lines.

In MOR- HEK cells, the concentration-response effects of BE 1–11, BE 1–13, BE 1–17, and BE 1–20 were not significantly different from that of BE 1–31 which suggests that biotransformation may provide a complex mileu of MOR agonists. Albeit, the efficacy of BE 1–9 was significantly lower than those of other N-terminal fragments and furthermore the extent of inhibition was consistent with being a partial agonist at the MOR. These results were consistent with the studies of *Jaba et al.* in which they demonstrated that the peptide interaction with MOR varies with the length of opioid peptide and the analgesic potency of opioid peptides is proportional to their peptide lengths [47].

DOR is an opioid receptor that is also involved in mediating analgesia in inflammatory pain. We studied the concentration-response effects of BE 1–9, BE 1–11, BE 1–13, BE 1–17, BE 1–20, BE 1–31 on modulation of cAMP levels in DOR-HEK cells. All of the selected fragments inhibited the production of cAMP at concentrations of 1 µM. The results showed that, in contrast with the effect of BE 1–31 fragments in MOR-HEK cells, cAMP inhibition potencies did not decrease correspondingly with the decrease in length of N-terminal fragments in DOR-HEK cells. To confirm the activity of BE 1–31 and its metabolites on DOR, naloxone was used as an antagonist to block DOR. Our results confirmed that all of examined N-terminal BE 1–31 fragments are capable of activating DOR and thereby reduce cAMP production. However, unlike the potent effects of BE1–31 and its fragments on MOR-HEK and DOR-HEK, such fragments displayed significantly lower efficacy and potency in KOR-HEK cells (1000 times less effective than a selective KOR agonist, dynorphin A 1–17; data not shown), and therefore concentration-response analysis for these fragments were not performed.

Given the limited stability of BE in inflamed tissue, its analgesic effects may be due in part to the biotransformation fragments. The identification of BE 1–31 fragments and study of their effects demonstrates the role of tissue biotransformation in analgesia and that of opioid peptide fragments in analgesia in inflammatory pain.

## Supporting Information

Figure S1 **The effect of different concentrations of BE 1–9, BE 1–11, BE 1–13, BE 1–17, BE 1–20, BE 1–31, and fentanyl on cAMP inhibition in HEK 293 cells expressing MOR (0.3 nM to 1 µM).** HEK 293 cells expressing MOR (20000 cells/well) were used to investigate the effect of BE 1–31 and fragments on activation of MOR by measuring the level of cAMP by an alphascreen cAMP assay. FSK (100 µM) was used to stimulate the production of cAMP. Values represent mean ± SEM of at least three independent experiments. Concentration-response curves were plotted using one-site curve fitting in the Prism software using nonlinear regression analysis tools in Prism.
(DOCX)

Figure S2 **The effect of different concentrations of BE 1–9, BE 1–11, BE 1–13, BE 1–17, BE 1–20, BE 1–31, and SNC80 on cAMP inhibition in HEK 293 cells expressing DOR (0.3 nM to 1 µM).** HEK 293 cells expressing DOR (20000 cells/well) were used to investigate the effect of BE 1–31 and its fragments on activation of DOR by measuring the level of cAMP. FSK (50 µM) was used to stimulate the production of cAMP. Concentration-response curves were plotted using one-site curve fitting in the Prism software using nonlinear regression analysis tools in Prism.
(DOCX)

Table S1 **BE 1–31 fragments produced in inflamed tissue at pH 5.5, retention times, their corresponding observed mass/charge values, and the MRT and MRT relative for each fragments.**
(DOCX)

Table S2 **BE 1–17 fragments produced in inflamed tissue at pH 5.5, retention times, their corresponding observed mass/charge values, and the MRT and MRT relative for each fragments.**
(DOCX)

Table S3 **BE 1–13 fragments produced in inflamed tissue at pH 5.5, retention times, their corresponding observed mass/charge values, and the MRT and MRT relative for each fragments.**
(DOCX)

Table S4 **BE 1–11 fragments produced in inflamed tissue at pH 5.5, retention times, their corresponding observed mass/charge values, and the MRT and MRT relative for each fragments.**
(DOCX)
