# Supplementary material for: Beta-Endorphin 1–31 Biotransformation and cAMP Modulation in Inflammation
Source: PLoS One. 2014 Mar 11;9(3):e90380. doi: 10.1371/journal.pone.0090380 (PMC3949714; doi:10.1371/journal.pone.0090380)
Supplement: Figure S1 — The effect of different concentrations of BE 1–9, BE 1–11, BE 1–13, BE 1–17, BE 1–20, BE 1–31, and fentanyl on cAMP inhibition in HEK 293 cells expressing MOR (0.3 nM to 1 µM). HEK 293 cells expressing MOR (20000 cells/well) were used to investigate the effect of BE 1–31 and fragments on activation of MOR by measuring the level of cAMP by an alphascreen cAMP assay. FSK (100 µM) was used to stimulate the production of cAMP. Values represent mean ± SEM of at least three independent experiments. Concentration-response curves were plotted using one-site curve fitting in the Prism software using nonlinear regression analysis tools in Prism. (DOCX) [file pone.0090380.s001.docx]

**
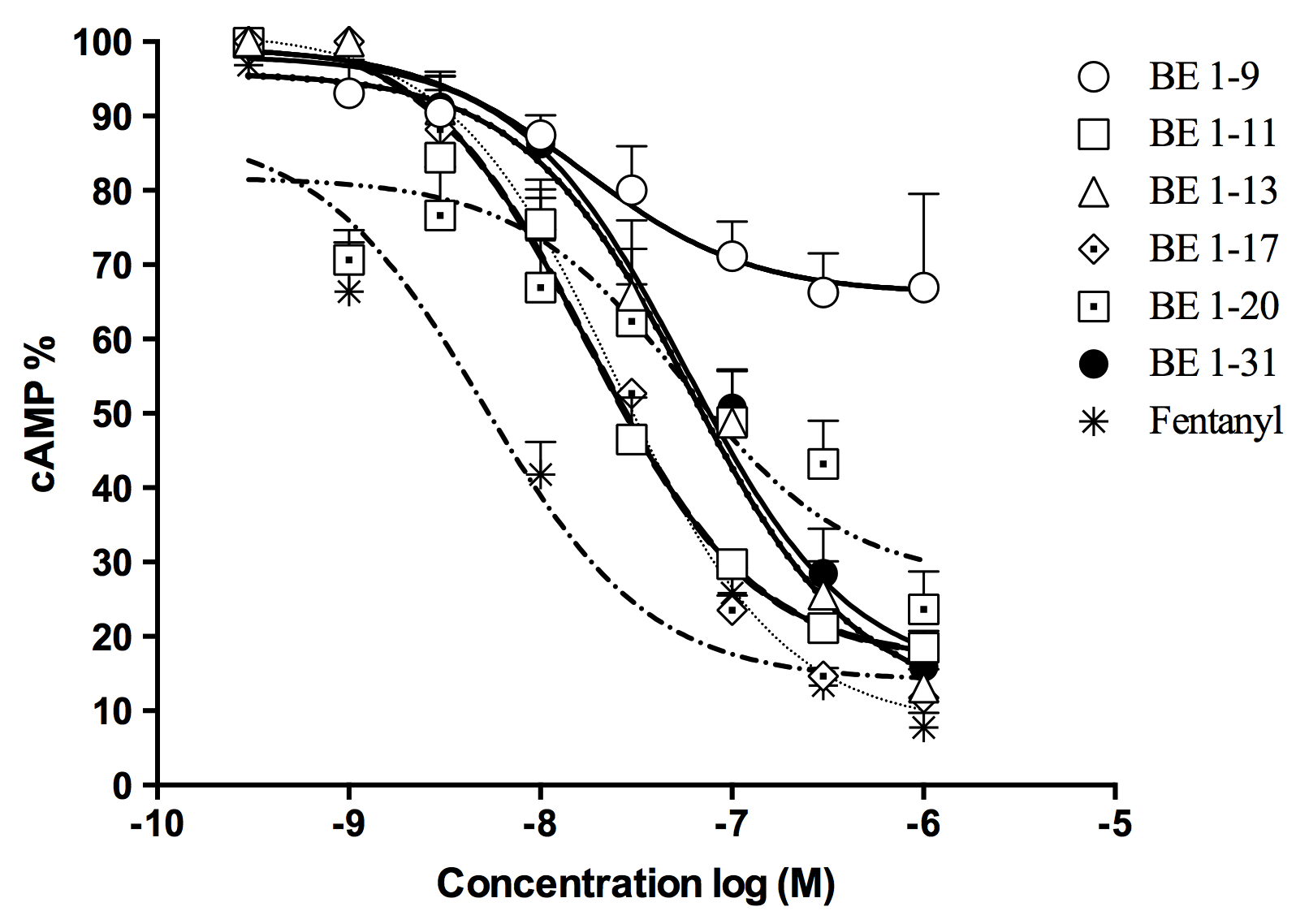
**

**Figure S1** **The effect of different concentrations of BE 1-9, BE 1-11, BE 1-13, BE 1-17, BE 1-20, BE 1-31, and fentanyl on cAMP inhibition in HEK 293 cells expressing MOR (0.3 nM to 1 µM).** HEK 293 cells expressing MOR (20000 cells/well) were used to investigate the effect of BE 1-31 and fragments on activation of MOR by measuring the level of cAMP by an alphascreen cAMP assay. FSK (100 μM) was used to stimulate the production of cAMP. Values represent mean ± SEM of at least three independent experiments.
