# Supplementary material for: Beta-Endorphin 1–31 Biotransformation and cAMP Modulation in Inflammation
Source: PLoS One. 2014 Mar 11;9(3):e90380. doi: 10.1371/journal.pone.0090380 (PMC3949714; doi:10.1371/journal.pone.0090380)
Supplement: Figure S2 — The effect of different concentrations of BE 1–9, BE 1–11, BE 1–13, BE 1–17, BE 1–20, BE 1–31, and SNC80 on cAMP inhibition in HEK 293 cells expressing DOR (0.3 nM to 1 µM). HEK 293 cells expressing DOR (20000 cells/well) were used to investigate the effect of BE 1–31 and its fragments on activation of DOR by measuring the level of cAMP. FSK (50 µM) was used to stimulate the production of cAMP. Concentration-response curves were plotted using one-site curve fitting in the Prism software using nonlinear regression analysis tools in Prism. (DOCX) [file pone.0090380.s002.docx]

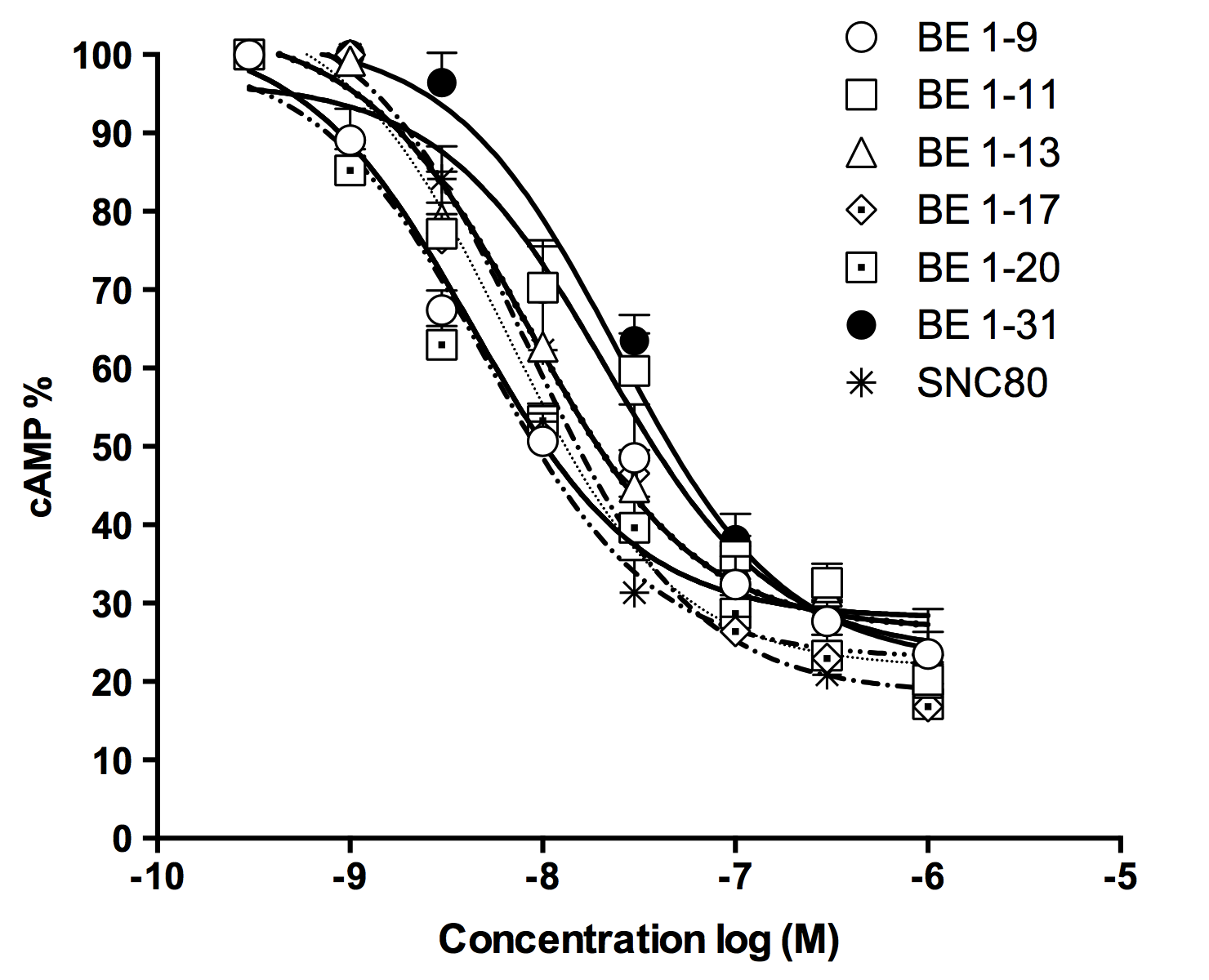


**Figure S2** **The effect of different concentrations of BE 1-9, BE 1-11, BE 1-13, BE 1-17, BE 1-20, BE 1-31, and SNC80 on cAMP inhibition in HEK 293 cells expressing DOR** (0.3 nM to 1 µM). HEK 293 cells expressing DOR (20000 cells/well) were used to investigate the effect of BE 1-31 and its fragments on activation of DOR by measuring the level of cAMP. FSK (50 μM) was used to stimulate the production of cAMP.. Concentration-response curves were plotted using one-site curve fitting in the Prism software using nonlinear regression analysis tools in Prism.
