# Supplementary material for: Beta-Endorphin 1–31 Biotransformation and cAMP Modulation in Inflammation
Source: PLoS One. 2014 Mar 11;9(3):e90380. doi: 10.1371/journal.pone.0090380 (PMC3949714; doi:10.1371/journal.pone.0090380)
Supplement: Table S1 — BE 1–31 fragments produced in inflamed tissue at pH 5.5, retention times, their corresponding observed mass/charge values, and the MRT and MRT relative for each fragments. (DOCX) [file pone.0090380.s003.docx]

**Table S1** BE 1-31 fragments produced in inflamed tissue at pH 5.5, retention times, their corresponding observed mass/charge values, and the MRT and MRT relative for each fragments.

| RT (min) | Metabolites | Observed mass/charge value | | |  |  | MRT | MRT relative |
| --- | --- | --- | --- | --- | --- | --- | --- | --- |
|  |  | [M+H]^+1^ | [M+H]^+2^ | [M+H]^+3^ | [M+H]^+4^ | [M+H]^+5^ |  |  |
| 5.4 | BE 29-31 | 332.5 | _a | 83.2 | _a | _a | _b | _b |
| 13.67 | BE 19-31 | 1478.5 | 740.2 | 494 | 370.6 | _a | 80 | 27.5 |
| 14.3 | BE 19-30 | 1350.7 | 676.3 | 451.2 | _a | _a | 72.2 | 24.9 |
| 14.3 | BE 20-29 | 1165.6 | 583.6 | 389.5 | _a | _a | 8.4 | 2.9 |
| 14.3 | BE 20-31 | 1350.7 | 676.3 | 451.2 | _a | _a | 72.2 | 24.9 |
| 16.1 | BE 20-24 | 558.6 | 279.6 | _a | _a | _a | 98 | 33.8 |
| 16.1 | BE 20-27 | 909 | 455.4 | 303.7 | _a | _a | 97.1 | 33.5 |
| 16.1 | BE 20-28 | 1037.2 | 519.2 | 260 | _a | _a | 87.6 | 30.2 |
| 16.1 | BE 18-31 | 1626.6 | 813.9 | 542.9 | 407.6 | 326 | 18.1 | 6.2 |
| 17.5 | BE 2-11 | 1072.1 | 537 | _a | _a | _a | 37 | 12.7 |
| 18.38 | BE 2-9 | 857.1 | 429.2 | _a | _a | _a | 20 | 7 |
| 18.4 | BE 6-16 | 1192.2 | 595.6 | 297.3 | _a | _a | 22.4 | 7.7 |
| 19 | BE 5-24 | 1125.9 | 750.9 | _a | _a | _a | 25.1 | 8.7 |
| 19 | BE 10-16 | 745.7 | 373.3 | _a | _a | _a | 44.3 | 15.3 |
| 19.96 | BE 2-13 | 1270.2 | 636 | _a | _a | _a | 65 | 22.4 |
| 19.96 | BE 1-11 | 1236.4 | 619.2 | 412.9 | 310 | 205 | 57.8 | 20 |
| 20.2 | BE 1-9 | 1020.1 | 511.1 | _a | _a | _a | 11.4 | 3.9 |
| 20.9 | BE 1-13 | 1433.6 | 717.4 | 478.7 | _a | _a | 44 | 15.2 |
| 22.2 | BE 2-14 | 1383.4 | 692.3 | 462.1 | _a | _a | 25.1 | 8.6 |
| 22.43 | BE 5-18 or 4-17 | 1583.9 | 792.7 | 528.6 | _a | _a | 19.1 | 6.6 |
| 22.44 | BE 15-24 | 1148.1 | 574.5 | _ | _a | _a | 25.2 | 8.7 |
| 23.2 | BE 1-16 | 1746.6 | 874 | 583.1 | _a | _a | 9.8 | 3.4 |
| 24.2 | BE 1-15 | 1647.7 | 823.4 | 549.4 | _a | _a | 12 | 4.1 |
| 24.5 | BE 1-17 | 1859.6 | 930.2 | 620.8 | _a | _a | 25 | 8.6 |
| 24.7 | BE 6-31 | _a | 1456.2 | 971.3 | 583.2 | _a | 40 | 13.8 |
| 24.9 | BE 10-31 | _a | 1233.9 | 823.1 | 617.4 | 494.1 | 29 | 10 |
| 25.2 | BE 1-20 | _a | 1126.7 | 751 | 563.6 | 450.8 | 34.2 | 11.8 |
| 25.8 | BE 1-31 | _a | 1734.1 | 1156.7 | 868 | 694.2 | 2.9 | 1 |
| 26.9 | BE 10-28 | _a | 1077 | 718.2 | 539.1 | 431.3 | 32.3 | 11.1 |
| 28.2 | BE 10-18 | 1006.3 | 503.4 | _a | _a | _a | 13.3 | 4.6 |
| 28.2 | BE 10-24 | _a | 837.3 | 558.8 | _a | _a | 11.3 | 3.9 |

-^a^ Not detected, -^b^ Not calculated.
