# Supplementary material for: Beta-Endorphin 1–31 Biotransformation and cAMP Modulation in Inflammation
Source: PLoS One. 2014 Mar 11;9(3):e90380. doi: 10.1371/journal.pone.0090380 (PMC3949714; doi:10.1371/journal.pone.0090380)
Supplement: Table S2 — BE 1–17 fragments produced in inflamed tissue at pH 5.5, retention times, their corresponding observed mass/charge values, and the MRT and MRT relative for each fragments. (DOCX) [file pone.0090380.s004.docx]

**Table S2** BE 1-17 fragments produced in inflamed tissue at pH 5.5, retention times, their corresponding observed mass/charge values, and the MRT and MRT relative for each fragments.

| RT (min) | Metabolites | Observed mass/charge value | | | MRT | MRT relative |
| --- | --- | --- | --- | --- | --- | --- |
|  |  | [M+H]^+1^ | [M+H]^+2^ | [M+H]^+3^ |  |  |
| 14.62 | BE 6-14 | 991.1 | 496.5 | _a | _b | _b |
| 14.92 | BE 7-14 | 890 | 445.6 | _a | _b | _b |
| 15.52 | BE 2-11 | 1072.1 | 537 | _a | 31.8 | 5.7 |
| 15.67 | BE 4-13 | 1157 | 578.7 | _a | _b | _b |
| 15.87 | BE 2-9 | 856.8 | 429.3 | _a | 21 | 3.7 |
| 16 | BE 6-16 | 1191.3 | 596.4 | _a | _b | _b |
| 16 | BE 7-16 | 1089.9 | 546.1 | _a | _b | _b |
| 16.27 | BE 8-14 | 800.9 | 401.2 | _a | _b | _b |
| 16.92 | BE 5-16 | 1321.9 | 662.1 | _a | 13.2 | 2.4 |
| 17.08 | BE 2-13 | 1270.1 | 635.8 | _a | 25.5 | 4.5 |
| 17.58 | BE 1-11 | 1235.4 | 618.5 | _a | 17.2 | 3.1 |
| 18.13 | BE 1-9 | 1019.8 | 510.9 | _a | 28 | 5 |
| 18.68 | BE 4-14 | 1269.4 | 635.6 | 424 | _b | _b |
| 18.78 | BE 1-13 | 1434.6 | 717.4 | _a | 21.7 | 3.9 |
| 19.32 | BE 4-16 | 1469.3 | 735.4 | 490.8 | _b | _b |
| 20.1 | BE 2-14 | 1383.4 | 692.5 | 462.1 | 18.9 | 3.4 |
| 20.1 | BE 3-14 | 1326.3 | 663.9 | 443 | _b | _b |
| 20.13 | BE 4-15 | 1383.2 | 692.3 | 462.1 | _b | _b |
| 20.43 | BE 2-16 | 1583.7 | 792.7 | _a | 15.1 | 2.7 |
| 20.43 | BE 3-16 | 1526.2 | 764.3 | 510 | 11 | 2 |
| 20.53 | BE 3-12 | 1114.4 | 557.8 | _a | _b | _b |
| 21.13 | BE 1-14 | 1546.4 | 773.5 | 516.5 | 13.8 | 2.5 |
| 21.33 | BE 2-15 | 1482.2 | 741.6 | 495.1 | _b | _b |
| 21.38 | BE 1-16 | 1746.6 | 873.9 | 583.2 | 7.6 | 1.4 |
| 21.48 | BE 3-11 | 1013 | 507.6 | _a | _b | _b |
| 22.33 | BE 1-15 | 1645.5 | 823.6 | 549.4 | 8.3 | 1.5 |
| 24 | BE 3-17 | 1639.6 | 820.6 | 547.5 | 6.2 | 1.1 |
| 24 | BE 2-17 | 1696.6 | 849.2 | 566.5 | 7.45 | 1.3 |
| 24.3 | BE 1-17 | 1859.7 | 930 | 620.9 | 5.6 | 1 |

-^a^ Not detected, -^b^ Not calculated.
