# Supplementary material for: Beta-Endorphin 1–31 Biotransformation and cAMP Modulation in Inflammation
Source: PLoS One. 2014 Mar 11;9(3):e90380. doi: 10.1371/journal.pone.0090380 (PMC3949714; doi:10.1371/journal.pone.0090380)
Supplement: Table S3 — BE 1–13 fragments produced in inflamed tissue at pH 5.5, retention times, their corresponding observed mass/charge values, and the MRT and MRT relative for each fragments. (DOCX) [file pone.0090380.s005.docx]

**Table S3** BE 1-13 fragments produced in inflamed tissue at pH 5.5, retention times, their corresponding observed mass/charge values, and the MRT and MRT relative for each fragments

| Rt (min) | Metabolites | Observed mass/charge values | | | MRT | MRT relative |
| --- | --- | --- | --- | --- | --- | --- |
|  |  | [M+H]^+1^ | [M+H]^+2^ | [M+H]^+3^ |  |  |
| 15.7 | BE 2-11 | 1072 | 537 | _a | 20.2 | 2.4 |
| 15.7 | BE 4-13 | 1156.2 | 579.1 | _a | 18 | 2.1 |
| 15.8 | BE 2-9 | 857 | 429.3 | _a | 19.8 | 2.4 |
| 16.2 | BE 2-12 | 1173.2 | 587.1 | _a | 18.1 | 2.2 |
| 17.2 | BE 2-13 | 1270.2 | 635.8 | _a | 16.4 | 2 |
| 17.2 | BE 3-13 | 1213.1 | 607.2 | _a | 16.1 | 2 |
| 17.4 | BE 1-11 | 1235.2 | 618.6 | 411.5 | 10.5 | 1.3 |
| 18.5 | BE 1-13 | 1433.5 | 717.5 | 478.5 | 8.3 | 1 |

-^a^ Not detected.
