# Supplementary material for: Beta-Endorphin 1–31 Biotransformation and cAMP Modulation in Inflammation
Source: PLoS One. 2014 Mar 11;9(3):e90380. doi: 10.1371/journal.pone.0090380 (PMC3949714; doi:10.1371/journal.pone.0090380)
Supplement: Table S4 — BE 1–11 fragments produced in inflamed tissue at pH 5.5, retention times, their corresponding observed mass/charge values, and the MRT and MRT relative for each fragments. (DOCX) [file pone.0090380.s006.docx]

**Table S4** BE 1-11 fragments produced in inflamed tissue at pH 5.5, retention times, their corresponding observed mass/charge values, and the MRT and MRT relative for each fragments.

| Rt (min) | Metabolites | Observed mass/charge values | | | MRT | MRT relative |
| --- | --- | --- | --- | --- | --- | --- |
|  |  | [M+H]^+1^ | [M+H]^+2^ | [M+H]^+3^ |  |  |
| 13.8 | BE 4-11 | 957.6 | 479.7 | _a | 22.7 | 2.1 |
| 15.4 | BE 3-11 | 1014.8 | 508.4 | _a | 14 | 1.3 |
| 15.7 | BE 2-11 | 1072 | 537 | _a | 15 | 1.4 |
| 15.8 | BE 2-9 | 857 | 429.3 | _a | 17 | 1.5 |
| 17.4 | BE 1-11 | 1235.2 | 618.6 | 411.5 | 11 | 1 |

-^a^ Not detected.
